# Prediction of gestational diabetes mellitus using machine learning from birth cohort data of the Japan Environment and Children's Study

**DOI:** 10.1038/s41598-023-44313-1

**Published:** 2023-10-13

**Authors:** Masahiro Watanabe, Akifumi Eguchi, Kenichi Sakurai, Midori Yamamoto, Chisato Mori, The Japan Environment Children’s Study (JECS) Group, Michihiro Kamijima, Shin Yamazakii, Yukihiro Ohya, Reiko Kishi, Nobuo Yaegashi, Koichi Hashimoto, Chisato Mori, Shuichi Ito, Zentaro Yamagata, Hidekuni Inadera, Takeo Nakayama, Tomotaka Sobue, Masayuki Shima, Seiji Kageyama, Narufumi Suganuma, Shoichi Ohga, Takahiko Katoh

**Affiliations:** 1 Department of Sustainable Health Science, Center for Preventive Medical Sciences, Chiba University, 1-33, Yayoicho, Inage-ku, Chiba, 263-8522 Japan; 2 Department of Nutrition and Metabolic Medicine, Center for Preventive Medical Sciences, Chiba University, Chiba, Japan; 3 Department of Bioenvironmental Medicine, Graduate School of Medicine, Chiba University, Chiba, Japan; 4 Nagoya City University, Nagoya, Japan; 5 National Institute for Environmental Studies, Tsukuba, Japan; 6 National Center for Child Health and Development, Tokyo, Japan; 7 Hokkaido University, Sapporo, Japan; 8 Tohoku University, Sendai, Japan; 9 Fukushima Medical University, Fukushima, Japan; 10 Yokohama City University, Yokohama, Japan; 11 University of Yamanashi, Chuo, Japan; 12 University of Toyama, Toyama, Japan; 13 Kyoto University, Kyoto, Japan; 14 Osaka University, Suita, Japan; 15 Hyogo Medical University, Nishinomiya, Japan; 16 Tottori University, Yonago, Japan; 17 Kochi University, Nankoku, Japan; 18 Kyushu University, Fukuoka, Japan; 19 Kumamoto University, Kumamoto, Japan

**Keywords:** Computational biology and bioinformatics, Endocrinology, Risk factors

## Abstract

Recently, prediction of gestational diabetes mellitus (GDM) using artificial intelligence (AI) from medical records has been reported. We aimed to evaluate GDM-predictive AI-based models using birth cohort data with a wide range of information and to explore factors contributing to GDM development. This investigation was conducted as a part of the Japan Environment and Children's Study. In total, 82,698 pregnant mothers who provided data on lifestyle, anthropometry, and socioeconomic status before pregnancy and the first trimester were included in the study. We employed machine learning methods as AI algorithms, such as random forest (RF), gradient boosting decision tree (GBDT), and support vector machine (SVM), along with logistic regression (LR) as a reference. GBDT displayed the highest accuracy, followed by LR, RF, and SVM. Exploratory analysis of the JECS data revealed that health-related quality of life in early pregnancy and maternal birthweight, which were rarely reported to be associated with GDM, were found along with variables that were reported to be associated with GDM. The results of decision tree-based algorithms, such as GBDT, have shown high accuracy, interpretability, and superiority for predicting GDM using birth cohort data.

## Introduction

Gestational diabetes mellitus (GDM) is the glucose intolerance that is first recognized during pregnancy. GDM is a common complication during pregnancy, affecting up to 15% of pregnant women worldwide, affecting both maternal and fetal status, and causing perinatal complications, including stillbirth, premature delivery, macrosomia, fetal hyperinsulinemia, and clinical neonatal hypoglycemia^1^. Fetuses exposed to a hyperglycemic environment have a higher risk of developing chronic diseases, such as obesity, diabetes, and cardiovascular disease^2–5^. Mothers are more likely to be diagnosed with GDM at 24–28 weeks of gestation. However, early interventions for GDM are effective in reducing its impact on mothers and fetuses^6^.

Diagnostic technology using artificial intelligence (AI) for disease evaluations has shown the equivalent performance to that of clinicians, depending on the intended use and combination of AI algorithms^7^. These results come from the application of several characteristic types of AI algorithms. For example, deep learning is effective for unstructured data (e.g., images or sound data)^8^. Actually, deep learning has a variety of training models in the fields of image, natural language, and audio data. However, Grinsztajn et al. reported that they are inferior to decision tree-based models for tabular data^9^. Because of problems with the interpretability of the model and results, this study considered other methods.

Other algorithms included support vector machine (SVM), random forest (RF), and the gradient boosting decision tree (GBDT), which are effective for database and other structured data^10–12^. These diagnostic technologies predict GDM with high accuracy^13–15^. However, the related studies used medical records as their data source^16^. Medical records provide accurate medical histories and conditions along with several blood test results obtained during pregnancy^13,14^. In contrast, birth cohort data, including information on lifestyle and living environment that is not typically included in medical records, are used for only a few AI studies examining the accuracy and efficacy of GDM prediction^15,16^. Birth cohort data include lifestyle and social factors, depending on the purpose of the survey^17,18^. A recent study reported that the modifiable risk factors for GDM during pregnancy include excessive weight gain, lifestyle behaviors, and poor mental health^19^. Analyzing a combination of medical records and lifestyle or living environment data can provide comprehensive evaluations of GDM prediction. Additionally, if lifestyle and living environment have a high impact on predictive accuracy, interventions targeting these factors or diagnostic algorithms considering these parameters may lead to early GDM prediction and prevention, thereby reducing GDM exacerbation and morbidity. Recently, a systematic review of the relationship between diet and physical activity and GDM has been reported^20^. In this report, the authors report that pre-pregnancy or early pregnancy intervention is effective in preventing GDM. This suggests that intervention can prevent GDM if it can be predicted at an early stage. For this purpose, it is important to investigate whether there are other factors in addition to the ones that are already reported that are predictive of GDM. The Japan Environment and Children’s Study (JECS) (2011–2014), a large birth cohort study, recruited participants from 15 Regional Centres in Japan. It enrolled pregnant women and collected a wide range of information (e.g., living environment and lifestyle factors) to reveal the environmental factors affecting children’s health and development^17,18^. Investigations using the JECS data have shown significant associations between GDM and various specific lifestyle factors (e.g. social capital)^21^. These were the projects that examined the relationship between limited variables and GDM. However, no study involved AI application to assess the relationship between GDM and comprehensive maternal data from a large cohort. Therefore, we aimed to evaluate the accuracy, predictive value, and utility of each AI algorithm in developing a GDM prediction model, and explore the factors contributing to GDM development using structured data, various parameters, and large data from the JECS.

## Results

Of the 103,060 JECS cohort participants, excluding those not suitable for analysis, there were 82,698 participants. Of these, 624 were GDM in groups with a past history of GDM (GDM-PH(+)) and 82,074 were GDM in groups without a past history of GDM (GDM-PH(−)). Table 1 shows characteristics of the pregnant women. The incidence of GDM according to the study data was 2.7%. The incidence of GDM-PH(+) and GDM-PH(−) was 52.3% and 2.7%, respectively. The number of weeks at the time of completion of the Maternal questionnaire at study enrollment for GDM-PH(+) and GDM-PH(−) was 14.88 weeks and 13.99 weeks, respectively.

**Table 1:** Baseline characteristics of the study population.

	All		GDM-PH(+)		GDM-PH(−)	
Number	82,698		624		82,074	
Number of weeks at time of completion of maternal questionnaire at study enrollment(weeks) (mean, SD)	13.99	4.26	14.89	3.54	13.99	4.26
Age (year) (mean, SD)	30.77	4.97	32.93	4.66	30.76	4.96
Height (cm) (mean, SD)	158.13	5.35	157.41	5.39	158.14	5.35
Pre-pregnancy weight (kg) (mean, SD)s	53.12	8.84	60.42	13.85	53.06	8.77
Pre-pregnancy BMI (kg/m^2^) (mean, SD)	21.23	3.29	24.35	5.33	21.20	3.25
Maternal active smoking during pregnancy						
Never (N, %)	48,302	58.41	317	50.80	47,985	58.47
Previously did, but quit before realizing current pregnancy (N, %)	19,637	23.75	204	32.69	19,433	23.68
Previously did, but quit after realizing current pregnancy (N, %)	10,541	12.75	68	10.90	10,473	12.76
Currently smoking (N, %)	3670	4.44	30	4.81	3640	4.44
Maternal drinking during pregnancy						
Never (N, %)	28,720	34.73	227	36.38	28,493	34.72
Previously did (N, %)	45,659	55.21	324	51.92	45,335	55.24
Currently drinking (N, %)	8012	9.69	71	11.38	7941	9.68
Educational background						
Junior high school (N, %)	3610	4.46	38	6.19	3572	4.44
High school (N, %)	25,230	31.15	216	35.18	25,014	31.12
Technical junior college (N, %)	1317	1.63	16	2.61	1301	1.62
Technical/vocational college (N, %)	18,772	23.18	124	20.20	18,648	23.20
Associate degree (N, %)	14,385	17.76	101	16.45	14,284	17.77
Bachelor’s degree (N, %)	16,483	20.35	107	17.43	16,376	20.37
Graduate degree (N, %)	1192	1.47	12	1.95	1180	1.47
GDM during pregnancy (N, %)	2253	2.72	307	49.20	1946	2.37

*BMI* body mass index, *GDM* gestational diabetes mellitus, *GDM-PH(+)* past history of GDM, *GDM-PH(−)* no past history of GDM, *SD* standard deviation.

The results of comparing the predictive accuracy of each algorithm are shown in Table 2. In the SVM model, overfitting occurred in both datasets; these overfitted models classified all input data as non-GDM. The results of the RF and LR models for the GDM-PH(+) group showed an area under the receiver operating characteristic curve (AUC) of 0.52 (95% CI 0.43–0.61) and 0.56 (95% CI 0.46–0.67), respectively. In the GBDT model for the GDM-PH(+) group, the performance were as follows: AUC = 0.67 (95% CI 0.57–0.75) and True Positive Rate (TPR) = 0.52 (95% CI 0.15–0.68). In contrast, the TPR of the RF and LR models for GDM-PH(−) mothers were zero due to overfitting. In the GBDT model for the GDM-PH(−) group, the performance were as follows: AUC = 0.74 (95% CI 0.71–0.77) and TPR = 0.01(95% CI 0.00–0.02).

**Table 2:** AUC, TPR, and FPR for the training dataset in various algorithms.

	GDM-PH(+) (N = 624)						GDM-PH(−) (N = 82,074)					
	AUC		TPR (%)		FPR (%)		AUC		TPR (%)		FPR (%)	
	Mean	95% CI	Mean	95% CI	Mean	95% CI	Mean	95% CI	Mean	95% CI	Mean	95% CI
SVM	0.51	(0.45–0.59)	0.37	(0.00–1.00)	0.36	(0.09–0.98)	0.50	(0.50–0.50)	0.0	0.0	0.0	0.0
RF	0.52	(0.43–0.61)	0.46	(0.30–0.62)	0.42	(0.26–0.59)	0.50	(0.50–0.50)	0.0	0.0	0.0	0.0
GBDT	0.67	(0.57–0.75)	0.52	(0.15–0.68)	0.37	(0.15–0.52)	0.74	(0.71–0.77)	0.01	(0.00–0.02)	0.0	0.0
LR	0.56	(0.46–0.67)	0.55	(0.40–0.70)	0.45	(0.30–0.60)	0.67	(0.64–0.70)	0.0	0.0	0.0	0.0

*AUC* area under the receiver operating characteristic curve, *CI* confidence interval, *GDBT* gradient boosting decision tree, *GDM* gestational diabetes mellitus, *GDM-PH(+)* past history of GDM, *GDM-PH(−)* no past history of GDM, *LR* logistic regression, *RF* random forest, *SVM* support vector machine.

For the GDM-PH(−) group, wherein overfitting occurred frequently, the results of changing the sampling methods are shown in Table 3. The results obtained using the SVM model did not change, even after altering the sampling methods. However, the results of undersampling in the RF model improved; the TPR increased to 0.18 (95% CI 0.14–0.22). The TPR also improved in both undersampling and oversampling in the GBDT and LR models as follows: undersampling in GBDT, 0.35 (95% CI 0.34–0.38); oversampling in GBDT, 0.21 (95% CI 0.16–0.27); undersampling in LR, 0.24 (0.17–0.30); and oversampling in LR, 0.23 (0.17–0.28). Following changes in sampling methods, undersampling showed higher accuracy than oversampling in the GBDT, LR, and RF models (except the SVM models).

**Table 3:** Result of resampling for the GDM-PH(−) group.

	AUC		TPR (%)		FPR	
	Mean	95% CI	Mean	95% CI	Mean	95% CI
GDM-PH(−) (N = 82,074)						
SVM						
Undersampling	0.50	(0.50–0.50)	0.00	(0.00–0.00)	0.00	(0.00–0.00)
Oversampling	0.50	(0.50–0.50)	0.00	(0.00–0.00)	0.00	(0.00–0.00)
RF						
Undersampling	0.57	(0.55–0.59)	0.18	(0.14–0.22)	0.04	(0.04–0.05)
Oversampling	0.50	(0.50–0.50)	0.00	(0.00–0.00)	0.00	(0.00–0.00)
GBDT						
Undersampling	0.64	(0.63–0.65)	0.35	(0.34–0.38)	0.08	(0.07–0.10)
Oversampling	0.59	(0.57–0.62)	0.21	(0.16–0.27)	0.03	(0.02–0.04)
LR						
Undersampling	0.57	(0.55–0.60)	0.24	(0.17–0.30)	0.09	(0.07–0.11)
Oversampling	0.57	(0.55–0.60)	0.23	(0.17–0.28)	0.08	(0.06–0.10)

*AUC* area under the receiver operating characteristic curve, *CI* confidence interval, *GDBT* gradient boosting decision tree, *GDM* gestational diabetes mellitus, *GDM-PH(+)* past history of GDM, *GDM-PH(−)* no past history of GDM, *LR* logistic regression, *RF* random forest, *SVM* support vector machine.

**Figure 1:**
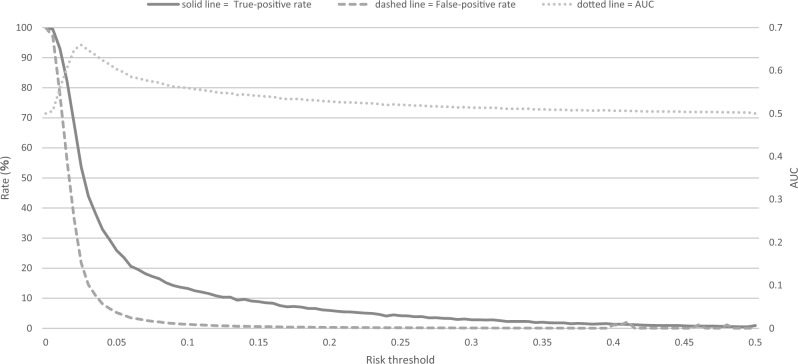
Changes in true-positive rate and false positive rate by differences in risk thresholds in GBDT. *AUC* area under the receiver operating characteristic curve, *GDBT* gradient boosting decision tree.

Using GBDT modeling for GDM-PH(−) group, the relationship between TPR, false-positive rate (FPR), and change in AUC on altering the risk threshold is shown in Fig. 1. When the risk threshold was reduced, the TPR increased faster than the FPR. The AUC yielded a unimodal graph with a maximum value of 0.66 when the risk threshold was 0.025. In other models, the probability of GDM occurrence was zero in most input data due to overfitting; thus, altering the risk threshold was ineffective.

**Figure 2:**
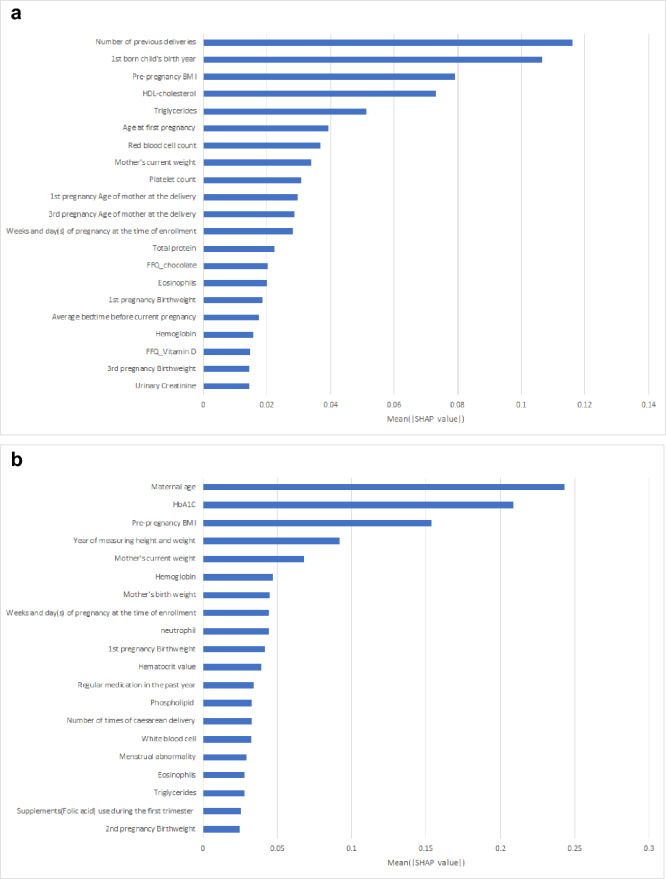
(**a**) SHAP summary plot for GDM-PH(+) (top 20 values) (**b**) SHAP summary plot for GDM-PH(−) (top 20 values). *BMI* body mass index, *GDM* gestational diabetes mellitus, *GDM-PH(+)* past history of GDM, *GDM-PH(-)* no past history of GDM.

Variables with high variable importance (VIP) identified in the analysis of the GBDT model without changing the sampling methods are shown in Table 4. Variables with high VIP in the GDM-PH(−) group included HbA1c levels, BMI before pregnancy, and maternal age. Variables with high VIP in the GDM-PH(+) group included triglyceride levels, platelet count, and firstborn child’s birth year. SHAP (SHapley Additive exPlanation) summary plot (Mean (|SHAP Value|) is shown in Fig. 2a. Variables with high Mean (|SHAP Value|) in the GDM-PH(+) group included number of previous deliveries, 1st born child's birth year, and BMI before pregnancy. Figure 2b shows variables with a high Mean (|SHAP Value|) in the GDM-PH(−) group, including maternal age, HbA1c levels, and BMI before pregnancy.

**Table 4:** Variable importance, top 20 in GBDT, and mean and SD of each variable.

VIP rank	GDM-PH(+)						GDM-PH(−)					
		N	GDM		Non-GDM			N	GDM		Non-GDM	
			Mean	SD	Mean	SD			Mean	SD	Mean	SD
1	Triglyceride (mg/dL)	608	157.3	63.4	141.7	58.9	HbA1c (%)	79,752	5.2	0.4	5.0	0.3
2	Platelet count (*10^4^/μL)	606	26.6	5.6	25.3	5.2	Pre-pregnancy BMI (kg/m^2^)	82,036	23.5	5.0	21.1	3.2
3	1st born child's birth year (year)	526	2007.9	3.8	2008.6	3.7	Maternal age (year)	82,055	33.1	4.9	30.7	5.0
4	HDL-cholesterol (mg/dL)	608	72.0	12.3	76.1	14.5	Mother's current weight (kg)	80,454	59.6	13.0	53.9	9.1
5	Number of previous deliveries (times)	620	1.1	0.9	1.4	0.9	Mother's birth weight (g)	72,919	3037.8	437.9	3091.8	415.7
6	Age at first pregnancy (year)	548	27.6	5.8	27.3	5.6	1st pregnancy birth weight (g)	37,548	3045.9	507.7	2982.4	441.6
7	1st pregnancy birth weight (g)	402	3055.0	549.1	3100.5	540.9	Triglyceride (mg/dL)	79,841	148.7	70.8	129.6	56.6
8	Red blood cell count (*10^4^/μL)	606	414.1	38.2	410.6	37.1	Platelet count (*10^4^/μL)	79,627	26.2	5.6	24.8	5.1
9	Pre-pregnancy BMI (kg/m^2^)	624	25.5	5.7	23.3	4.7	Phospholipid (mg/dL)	79,841	242.5	34.4	235.0	33.6
10	Hematocrit (%)	606	36.8	2.9	36.7	2.6	Hematocrit (%)	79,627	36.8	2.8	36.0	2.7
11	Hemoglobin (g/dL)	606	12.2	1.1	12.2	0.9	Mean cell hemoglobin (pg)	79,627	29.9	2.0	29.9	1.9
12	1st pregnancy Age of mother at the delivery	470	28.0	6.0	27.9	5.2	SF-8 MCS	80,708	46.1	7.3	46.0	7.3
13	Mother's current weight (kg)	616	63.0	14.2	58.5	12.1	SF-8 PCS	80,708	44.4	7.7	44.9	7.4
14	SF-8 MCS	613	45.7	7.4	46.5	7.0	Hemoglobin (g/dL)	79,627	12.3	1.0	12.0	1.0
15	Weeks of pregnancy at the time of enrollment (week)	384	13.8	3.4	12.9	3.1	Urinary creatinine (mg/dL)	79,822	106.3	66.5	100.2	62.1
16	White blood cell (/μL)	606	8403.3	1951.1	8044.1	2013.1	Monocyte (%)	79,626	4.7	1.3	4.8	1.3
17	Lymphocytes (%)	606	19.7	5.2	19.9	5.3	Eosinophil (/μL)	79,626	1.7	1.4	1.9	1.6
18	SF-8 PCS	613	44.5	7.1	44.5	7.8	Weeks of pregnancy at the time of enrollment	51,007	12.4	3.2	12.4	3.3
19	Mean corpuscular hemoglobin concentration (%)	606	33.0	1.1	33.2	1.0	Neutrophile (%)	79,626	74.5	5.6	73.9	6.2
20	Eosinophil (/μL)	606	1.6	1.2	1.8	1.3	Year of measuring height and weight	80,045	2012.4	0.9	2012.4	0.9

*BMI* body mass index, *GDBT* gradient boosting decision tree, *GDM* gestational diabetes mellitus, *GDM-PH(−)* no past history of GDM, *GDM-PH(+)* past history of GDM, *HDL*-*Cholesterol* High Density Lipoprotein-Cholesterol, *SD* standard deviation, *SF-8 MCS* SF-8 mental component summary, *SF-8 PCS* SF-8 physical component summary.

## Discussion

We compared four machine learning methods to improve GDM prediction models based on a large birth cohort. GBDT exhibited the highest accuracy, followed by LR, RF, and SVM. Without changing the sampling methods, overfitting occurred upon the use of all algorithms except for GBDT for GDM-PH(−). The accuracy for GDM prediction of all algorithms, except for SVM, improved without overfitting using undersampling or oversampling. Changing the risk thresholds improved the accuracy of GBDT.

Furthermore, GBDT results were more accurate than the existing method wherein LR used only maternal age, pre-pregnancy BMI, and laboratory results of specimens (see Supplementary Table S1 online). This could be because variables useful for GDM prediction can be increased using JCES data and GBDT can construct the boundary surface non-linearly.

There were some differences in variables important for predicting GDM in the GBDT model between the GDM-PH(+) (recurrent GDM) and GDM-PH(−) (new-onset GDM) groups. Thus, differences in VIP between recurrent GDM and new-onset GDM in JECS data were not based on parity.

The RF, GBDT, and SVM algorithms used are reportedly effective for structured data; thus, we compared them to determine the most appropriate one for the JECS data. For the GDM-PH(+) group, overfitting occurred in the data in the SVM model. Other algorithms yielded stable results without overfitting. For the GDM-PH(−) group, overfitting occurred in the data of all models, except for the GBDT model. Owing to the exploratory approach for predicting GDM, the data set used here was unique because it included many variables that do not affect GDM. Those noisy data cause compounding negative effects on generalizability and overfitting^22^. Imbalanced datasets often result in an overfitted model to achieve high classification accuracy^23^. The GDM-PH(+) group (N = 624) had a much smaller sample size than the GDM-PH(−) group (N = 82,074). Both groups included many variables that did not affect GDM. However, the ratio of the GDM and non-GDM groups were almost similar in the GDM-PH(+) group. Furthermore, the GDM-PH(−) group had a very low incidence of GDM (2.8%). In the SVM model, the problems related to many variables that do not improve the predictability negatively affect the analysis. SVM extracts records near the boundary surface as support vectors and creates a discrimination surface using only support vectors^10^. Thus, SVM can reduce the number of records used for analysis. However, SVM is not an algorithm to properly select variables from a large number of variables. Therefore, the choice of support vectors in our study was inappropriate, possibly leading to overfitting.

In contrast, RF and GBDT models use decision tree algorithms. The decision tree requires repeated binary decision-making. Therefore, variables that do not included the predictability of GDM are not included in the decision tree^11,12^. Therefore, decision tree algorithms are highly robust to data with many variables. In the RF algorithm, random sampling of the training dataset is performed as the first step to create multiple datasets^11^. Subsequently, the RF algorithm creates a decision tree model for each dataset to predict results by the majority rule. Sampling datasets from the GDM-PH(−) group using this particular algorithm may not ensure model diversity generated by random sampling, possibly leading to overfitting. In the GBDT model, hyperparameter optimization is performed using gradient descent before the start of each subsequent training session^12^. Therefore, unlike the RF model, the decision tree in the GBDT model may be trained while reducing the bias between the case and control groups. However, the TPR was not high even in the GBDT model (the only model without overfitting).

As in this study, the development of prediction models using data with a low case-to-control ratio requires adjustment of the sample size of the training data by changing the sampling method^24^. The TPRs of the RF, GBDT, and LR models were improved by changing the sampling method (Table 3). Undersampling could prevent overfitting with excessive control data. However, in the SVM model, the accuracy of GDM prediction has not improved, possibly because changes in sampling methods do not solve the problem of multidimensional data with many variables that do not improve the predictability. Considering oversampling, the TPR improved slightly in the GBDT and LR models, but overfitting occurred in the RF model. The oversampling technique randomly duplicates data until the case-to-control ratio reaches a specific value; thus, this technique may not solve the problem of the RF model (i.e., model diversity). In contrast, in the LR model, imbalance corrections, including changes in sampling methods may even worsen model performance^25^. In this study, the method of changing the risk threshold was used for imbalance corrections without changing the sampling method. The JECS data were not designed to estimate imbalance correction; thus, it was not possible to evaluate such effects in this study. However, our study demonstrated the potential to improve TPRs while maintaining the FPR low by changing the thresholds (Fig. 1). Generally, lowering the risk threshold increases both the TPR and FPR, but setting an appropriate risk threshold for LR and GBDT enables imbalance corrections without changing the sampling method. For setting the risk threshold, Goorbergh et al. used two fixed values—the prevalence of malignancy in the training dataset and the default risk threshold of 0.5. However, in this study, when the risk threshold was varied from 0 to 0.5 in steps of 0.005, the AUC reached its maximum value at 0.025, as did the GDM prevalence at 0.027.

We performed an exploratory analysis of factors contributing to GDM using AI. Typically, our exploratory methods have the following disadvantages: (1) the results may be inappropriate depending on the AI algorithms used, and (2) due to the cost, increasing the number of participants to obtain enough variables that are acceptable for the exploratory analysis was difficult. However, using sufficient data, selecting appropriate algorithms, and comparing VIPs, it was possible to identify variables previously not associated with GDM and verify previously reported associated factors.

In this study, we predicted the development of GDM based on information that could be collected in the early stages of pregnancy. Mothers are more likely to be diagnosed with GDM at 24–28 weeks of gestation. In this study, the average date of completion of the collected questionnaires was 14–15 weeks, which is considered early enough to predict the diagnosis, even if considering the time between the blood collection and the results of the tests. In meta-analysis, protective association of physical activity (21–46%) from GDM when comparing any type of physical activity to none in either the pre-pregnancy or early pregnancy period^20^. If a high-risk group near the 1st trimester can be extracted, it may lead to GDM prevention.

In this study, 775 questions were used to predict the incidence of GDM. Obviously, it would not be practical to build a prediction model using all of these questions, as it would take a lot of time to enter the predictors. Therefore, it is important to screen out as many variables as possible that are important for prediction. In this study, two evaluation criteria, VIP and Mean (|SHAP value|), were used to select predictors. High VIP variables identified in this study are listed in Table 4. Previous GDM studies identified a history of GDM in previous pregnancies, maternal age, and obesity as risk factors for GDM^26^. Additionally, the effect of GDM on the interpregnancy interval was reported^27^. The JECS data do not include the interpregnancy interval. Therefore, the firstborn child’s birth year was considered as an alternative variable. One study reported a significant difference in white blood cell count and platelet count between the GDM and non-GDM groups in the second trimester^28^. A meta-analysis showed a significant increase in lipid levels (e.g., triglyceride) in mothers with GDM in the first and second trimesters^29^. Variables that are reportedly associated with GDM in studies conducted before the JECS were also identified as factors with high VIP in this study. However, regarding urinary creatinine concentration, a study on the associations between urinary metals in early pregnancy and the subsequent risk of GDM reported no significant difference in urinary creatinine between GDM and non-GDM groups^30^. However, we revealed urinary creatinine concentration with a higher VIP from the GDM-PH(−) group, especially the nulliparous group. Although the reason for this is unknown, it may be a surrogate indicator for some other factor, such as physique.

The items in the questionnaire administered at enrollment in this study include the 8-item Short-Form Health Survey (SF-8) items for health-related quality of life (HRQOL)^31^. Physical component summary and mental component summary were variables with high VIP regardless of the presence or absence of a history of GDM. Regarding GDM and HRQOL, a systematic review examining the short- and long-term progression of HRQOL and their association with GDM diagnosis was reported; GDM does not directly lead to reduced QOL in mothers but causes some complicated interactions with psychological factors, resulting in reduced QOL^32^. The SF-8 data in this study were collected before 22 weeks of gestation. Our study results suggest that mothers’ HRQOLs are related to the risk of GDM; thus, GDM further reduces HRQOL.

Recent studies examining the association between mothers’ birth weights and GDM revealed that mothers with low birth weights or macrosomia were at higher risk of GDM^33^. We identified mothers’ birth weights as factors with high VIP in the GDM-PH(−) group. Hales et al. reported a correlation between low birth weight and subsequent glucose intolerance^34^. GDM is mild glucose intolerance; thus, mothers with low birth weights may have an increased risk of GDM.

High Mean|SHAP| variables identified in this study are shown Fig. 2a,b. Although similar variables to the VIPs were found in the top 20, SF-8 MCS and SF-8 PCS were absent from the top 20 for both GDM-PH(+) and GDM-PH(−).On the other hand, the GDM-PH(+) group showed a new variable, chocolate and vitamin D intake from the dietary questionnaire, and the GDM-PH(−) group showed a new variable, supplement intake (folic acid). Both vitamin D and folic acid have been reported to have an association with GDM^35,36^.

Although variables including those already reported to be associated with GDM, such as these, were detected in this study, the AUC score of gradient boosting for those with GDM history (0.67) was below the acceptable minimum for clinical implication (0.70). But in the JECS study, we are currently analyzing maternal genetic data, which will be provided in the future. By re-constructing the model after taking these genetic backgrounds into account, we expect to improve the prediction accuracy.

This study has some limitations. First, in Japan, the diagnostic criteria of the Japanese Society of Obstetrics and Gynecology are used to determine GDM. But the JECS is a multi-region, multi-medical institution cohort study; GDM data were obtained from medical record transcripts; thus, we could not review in detail the diagnostic criteria of GDM for the co-operating health care provider(s)^21^. Second, analysis in this study was performed considering information collected at the time of study enrollment. However, we did not consider the effects of other factors not identified in the JECS, especially genetic information and family history of diabetes mellitus. Third, information on diet was collected using self-administered questionnaires. Therefore, the results may not accurately reflect the actual food or nutrient intake. Fourth, the incidence of GDM in Japan is 7–13%^37^. However, the incidence of GDM in this study was 2.7%. This may indicate that the JECS included more health-conscious mothers or favored low enrollment for high-risk pregnancies, leading to sampling bias. Fifth, the JECS was conducted in Japan, and most participants were Japanese. Thus, generalization to populations from other countries may be inaccurate because the JECS results consider the unique living environment and lifestyle in Japan. Finally, with the size of the JECS data, it is difficult to obtain predictions by physicians as an external evaluation. Studies combining the findings of this study (mother's birth weight and psychological factors) with previously reported factors, including genes, are needed for more accurate prediction compared to prediction by other means, such as using genes.

In conclusion, we demonstrated that exploratory analysis using AI for a large birth cohort is possible through the appropriate use of algorithms. Algorithm comparison revealed high accuracy, interpretability, and superiority of decision tree-based algorithms, including GBDT considering datasets in this study. Further studies regarding GDM prediction using AI are needed to improve the TPR by collecting other variables, including genetic information and family history of diabetes mellitus. Using exploratory analysis of the JECS data, we identified the importance of previously reported variables related to GDM and new variables, such as HRQOL in early pregnancy and mothers’ birth weights related to GDM.

## Material and methods

### Data sources

The JECS is a nationwide birth cohort study. The design of the JECS study is described elsewhere^18^. The eligibility criteria for participants in the JECS did not consider the presence or absence of disease. This study used the jecs-ta-20190930 dataset, which was released in October 2019. The following data were identified from the dataset and used for analysis: maternal questionnaire data and dietary data from the survey administered at study enrollment (T1), medical record transcripts during pregnancy, child’s sex determined after delivery, laboratory results of specimens collected by 21 weeks of gestation, parental education and household income data collected during mid-late pregnancy, and parents’ birth weights (reported by the mother) after delivery. For the study outcome, GDM cases were defined as GDM during pregnancy per medical record transcripts. The maternal questionnaire included the Kessler Psychological Distress Scale (K6) as an indicator of psychological distress^38^, a short version of the International Physical Activity Questionnaire as an indicator of physical activity^39,40^, SF-8 Health Survey (SF-8) as an indicator of health-related quality of life^31^, and environmental exposures^41^. Food-intake and nutrient-intake data were obtained from the food frequency questionnaires validated in the Japan Public Health Center-based Prospective Study for the Next Generation^42^.

### Data preprocessing

**Figure 3:**
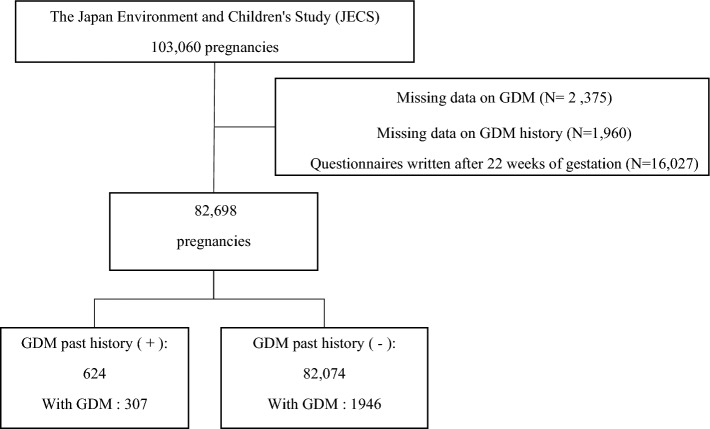
Flow chart showing the selection of the study population. *GDM* gestational diabetes mellitus.

The flow chart of the selection of participants is presented in Fig. 3. In this study, one pregnant woman with multiple fetuses is counted as one case. Of 103,060 pregnancies, the following were excluded from the present study: mothers with missing records on the diagnosis of GDM (N = 2375), mothers with a missing GDM history (N = 1960), and mothers who completed the T1 questionnaire at or after 22 weeks of gestation (N = 16,027). We followed 82,698 participants and observed 2253 pregnancies with GDM and 80,445 pregnancies without GDM. The recurrence rates of GDM were high^43^; thus, the remaining 82,698 pregnancies were divided as follows: (1) GDM in groups with a history of GDM (GDM-PH(+)) (N = 624) and (2) GDM in groups without a history of GDM (GDM-PH(−)) (N = 82,074).

As variables used to stratify this study, the history of GDM was reported in the T1 questionnaires. The qualitative text response data from open-ended questions and original variables summarized as separate variables (e.g., K6 and SF-8) were excluded from the analysis of the questionnaire data. Due to the very low number of triplets, information on multiple births was classified into singleton and multiple pregnancies. The variables used are shown in Supplemental Table S2. Non-ordinal categorical variables (e.g., marriage status, maternal occupation, infertility treatment status, and drugs used during pregnancy) were converted to dummy variables. BMI before pregnancy was calculated using weight before pregnancy as weight (kg)/height^2^ (m^2^). The variables reporting time of day with separate hours and minutes were converted to hours (i.e., hours + minutes/60). Continuous variables were normalized.

### Statistical analysis

The machine learning methods used in this study included SVM^10^ (which uses radial basis function [Gaussian] kernel), RF^11^, and GBDT^12^. LR^44^ was used as a reference.

All analyses were performed using python 3.8.5 in Jupyter Nortbook (Project Jupyter). Among the python libraries, scikit-learn 1.0.2 was used in the LR, SVM, and RF models; lightgbm 3.1.1 was used in the GBDT model; and imbalanced-learn 0.9.0 was used in undersampling and oversampling. The settings of the hyperparameters of these algorithms are shown in Table 5. SVM and LR do not accept datasets with missing data; thus, single imputation using mean substitution was performed. For cross-validation, our data were randomly divided into two groups with a ratio of 4:1, a training set, and a test set. To prevent overestimation from the use of imbalanced data, we used undersampling and oversampling methods, and the ratio of the GDM group to the non-GDM group was set at 1:2. True-positive rate (TPR), calculated as true positive/(true positive + false negative), and false-positive rate (FPR), calculated as false positive/(false positive + true negative), were the parameters of GDM prediction across the models. The AUC was used for evaluating model accuracy. The risk threshold for GDM classification was set at 0.5. Changes in the TPR and FPR when the risk threshold was changed from 0 to 0.5 step by 0.005 were examined.

**Table 5:** Settings of hyper parameters of each algorithm.

Algorithm	Parameters
SVM	C = 1.0, kernel = 'rbf', degree = 3, gamma = 'scale', coef0 = 0.0, shrinking = True, probability = False, tol = 0.001, cache_size = 200, class_weight = None, verbose = False, max_iter = -1, decision_function_shape = 'ovr', break_ties = False, random_state = None
RF	n_estimators = 40, *, criterion = 'gini', max_depth = 40, min_samples_split = 2, min_samples_leaf = 1, min_weight_fraction_leaf = 0.0, max_features = 'sqrt', max_leaf_nodes = None, min_impurity_decrease = 0.0, bootstrap = True, oob_score = False, n_jobs = None, random_state = None, verbose = 0, warm_start = False, class_weight = None, ccp_alpha = 0.0, max_samples = None
GBDT	'boosting': 'gbdt','application': 'binary','learning_rate': 0.03,'min_data_in_leaf': 10,'feature_fraction': 0.7,'num_leaves': 40,'metric': 'auc','drop_rate': 0.10
LR	–

*GDBT* gradient boosting decision tree, *LR* logistic regression, *RF* random forest, *SVM* support vector machine.

### Ethical approval

The JECS protocol was reviewed and approved by the Ministry of the Environment's Institutional Review Board on Epidemiological Studies (approval no.: 100910001) and the Ethics Committees of all participating institutions; the Medical Support Centre (National Centre for Child Health and Development), and 15 Regional Centers (Hokkaido University, Tohoku University, Fukushima Medical University, Chiba University, Yokohama City University, University of Yamanashi, University of Toyama, Nagoya City University, Kyoto University, Osaka University, Hyogo College of Medicine, Tottori University, Kochi University, University of Occupational and Environmental Health, and Kumamoto University). This study was conducted in accordance with the Helsinki Declaration and other nationally valid regulations and guidelines. In the JECS, written informed consent was obtained from all participants.

### Supplementary Information

Supplementary Tables.

## Data Availability

Data were unsuitable for public deposition due to ethical restrictions and the legal framework of Japan. It is prohibited by the Act on the Protection of Personal Information (Act No. 57 of May 30, 2003, amendment on September 9, 2015) to publicly deposit data containing personal information. Ethical Guidelines for Medical and Health Research Involving Human Subjects enforced by the Japan Ministry of Education, Culture, Sports, Science and Technology and the Ministry of Health, Labour and Welfare also restrict the open sharing of epidemiologic data. All inquiries about access to data should be sent to: jecs-en@nies.go.jp. The person responsible for handling inquiries sent to this e-mail address is Dr. Shoji F Nakayama, JECS Programme Office, National Institute for Environmental Studies.
