# Supplementary material for: Prediction of gestational diabetes mellitus using machine learning from birth cohort data of the Japan Environment and Children's Study
Source: Sci Rep. 2023 Oct 13;13:17419. doi: 10.1038/s41598-023-44313-1 (PMC10575866; doi:10.1038/s41598-023-44313-1)
Supplement: Supplementary file 1 — Supplementary Tables. [file 41598_2023_44313_MOESM1_ESM.docx]

|  | Supplementary table S1. AUC, recall rate and false positive rate for testset in LR by using only mathers age, pre-pregnancy BMI, laboratory results of specimens | | | | | | | | | | | | |
| --- | --- | --- | --- | --- | --- | --- | --- | --- | --- | --- | --- | --- | --- |
|  | GDM-PH(+) (n=624) | | | | | |  | GDM-PH(-) (n = 82,074) | | | | | |
|  | AUC | | recall rate (%) | | false positive rate (%) | |  | AUC | | recall rate (%) | | false positive rate (%) | |
|  | mean | 95% CI | mean | 95% CI | mean | 95% CI |  | mean | 95% CI | mean | 95% CI | mean | 95% CI |
| LR | 0.59 | (0.51-0.66) | 0.57 | (0.44-0.70) | 0.39 | (0.27-0.52) |  | 0.5 | (0.50-0.51) | 0.01 | (0.00-0.01) | 0.00 | (0.00-0.00) |

AUC, area under the receiver operating characteristic curve; LR, logistic regression; GDM, gestational diabetes mellitus; GDM-PH(+), past history of GDM; GDM-PH(-), no past history of GDM; CI , confidence interval;

| Supplementary tableS2. The values list used for analysis in this study | |
| --- | --- |
| index | value |
| 1 | Gestational diabetes |
| 2 | Mother's height |
| 3 | Mother's weight before pregnancy |
| 4 | Mother's age at regestration |
| 5 | Multiple births |
| 6 | Child's sex |
| 7 | SF-8 General health |
| 8 | SF-8 Physical functioning |
| 9 | SF-8 Role physical |
| 10 | SF-8 Bodily pain |
| 11 | SF-8 Vitality |
| 12 | SF-8 Social functioning |
| 13 | SF-8 Mental health |
| 14 | SF-8 Role emotional |
| 15 | SF-8 Physical component summary |
| 16 | SF-8 Mental component summary |
| 17 | K6 score |
| 18 | IPAQ Total Physical Activity MET-minutes/day |
| 19 | IPAQ calories burned/day |
| 20 | Current marital status |
| 21 | Number of individuals living together |
| 22 | Individuals living together (Living alone) |
| 23 | Individuals living together (Husband/romantic partner) |
| 24 | Individuals living together (Child(ren)) |
| 25 | Individuals living together (Father) |
| 26 | Individuals living together (Mother) |
| 27 | Individuals living together (Father-in-law) |
| 28 | Individuals living together (Mother-in-law) |
| 29 | Individuals living together (Sibling(s)) |
| 30 | Individuals living together (Husband/romantic partner's sibling(s)) |
| 31 | Individuals living together (Other individuals) |
| 32 | Number of children living together |
| 33 | Number of siblings living together |
| 34 | Number of husband/romantic partner's siblings living together |
| 35 | Number of other individuals living together |
| 36 | 1st born child's gender |
| 37 | 1st born child's birth year |
| 38 | 1st born child's birth month |
| 39 | 2nd born child's gender |
| 40 | 2nd born child's birth year |
| 41 | 2nd born child's birth month |
| 42 | 3rd born child's gender |
| 43 | 3rd born child's birth year |
| 44 | 3rd born child's birth month |
| 45 | 4th born child's gender |
| 46 | 4th born child's birth year |
| 47 | 4th born child's birth month |
| 48 | 5th born child's gender |
| 49 | 5th born child's birth year |
| 50 | 5th born child's birth month |
| 51 | Height before being pregnant (cm) |
| 52 | Weight before being pregnant (kg) |
| 53 | Feeling when pregnancy was found out |
| 54 | Is this your first pregnancy? |
| 55 | Age at first pregnancy |
| 56 | Number of times of vaginal delivery |
| 57 | Number of times of caesarean delivery |
| 58 | Number of times of ectopic pregnancy |
| 59 | Number of times of spontaneous abortion |
| 60 | Number of times of repeated spontaneous abortion |
| 61 | Number of times of induced abortion |
| 62 | Age at first menstrual period |
| 63 | History of taking oral contraceptives |
| 64 | Number of years an oral contraceptive was taken |
| 65 | Infertility treatment before current pregnancy |
| 66 | Ovulation-inducing drug |
| 67 | Artificial insemination |
| 68 | In-vitro fertilization |
| 69 | Other infertility treatment |
| 70 | Intracytoplasmic sperm injection |
| 71 | Blastocyst implantation |
| 72 | Number of fertilized eggs returned in the womb for the infertility treatment |
| 73 | Past disease of heart, brain and blood vessels (anemia) |
| 74 | Past disease of heart, brain and blood vessels (hypertension) |
| 75 | Past disease of heart, brain and blood vessels (hyperlipidemia) |
| 76 | Past disease of heart, brain and blood vessels (cerebral apoplexy (brain hemorrhage, brain infarction or subarachnoid hemorrhage) |
| 77 | Past disease of heart, brain and blood vessels (myocardial infarction and angina pectoris) |
| 78 | Past disease of heart, brain and blood vessels (congenital heart disease) |
| 79 | Past disease of heart, brain and blood vessels (Kawasaki disease) |
| 80 | Past disease of heart, brain and blood vessels (pregnancy-induced hypertension) |
| 81 | Past disease of heart, brain and blood vessels (other) |
| 82 | Past allergy and disease of ear-nose-throat (bronchial asthma) |
| 83 | Past allergy and disease of ear-nose-throat (allergic rhinitis or pollinosis) |
| 84 | Past allergy and disease of ear-nose-throat (chronic sinusitis) |
| 85 | Past allergy and disease of ear-nose-throat (chronic otitis media) |
| 86 | Past allergy and disease of ear-nose-throat (atopic dermatitis) |
| 87 | Past allergy and disease of ear-nose-throat (allergic conjunctivitis) |
| 88 | Past allergy and disease of ear-nose-throat (food allergy) |
| 89 | Past allergy and disease of ear-nose-throat (drug eruption and drug allergy) |
| 90 | Past allergy and disease of ear-nose-throat (hives) |
| 91 | Past allergy and disease of ear-nose-throat (contact dermatitis) |
| 92 | Past allergy and disease of ear-nose throat (sick-building syndrome) |
| 93 | Past allergy and disease of ear-nose-throat (multiple chemical sensitivity syndrome) |
| 94 | Past allergy and disease of ear-nose-throat (other) |
| 95 | Past disease of collagen and immunity (collagen disease) |
| 96 | Past disease of collagen and immunity (autoimmune disease) |
| 97 | Past disease of collagen and immunity (systemic lupus erythematosus) |
| 98 | Past disease of collagen and immunity (rheumatism) |
| 99 | Past disease of collagen and immunity (other diseases of collagen and immunity) |
| 100 | Past disease of endocrine system (type 1 diabetes) |
| 101 | Past disease of endocrine system (type 2 diabetes) |
| 102 | Past disease of endocrine system (gestational diabetes) |
| 103 | Past disease of endocrine system (hyperthyroidism or Graves' disease) |
| 104 | Past disease of endocrine system (hypothyroidism or Hashimoto’s thyroiditis) |
| 105 | Past disease of endocrine system (other diseases of endocrine system) |
| 106 | Past neurological/mental diseases (depression) |
| 107 | Past neurological/mental diseases (dysautonomia) |
| 108 | Past neurological/mental diseases (anxiety disorder (including anxiety neurosis, specific phobia, panic disorder, social phobia and obsessive-compulsive disorder)) |
| 109 | Past neurological/mental diseases (schizophrenia) |
| 110 | Past neurological/mental diseases (epilepsy) |
| 111 | Past neurological/mental diseases (migraine) |
| 112 | Past neurological/mental diseases (meningitis) |
| 113 | Past neurological/mental diseases (hydrocephalus) |
| 114 | Past neurological/mental diseases (other) |
| 115 | Past disease of digestive organs, liver and pancreas (reflux esophagitis) |
| 116 | Past disease of digestive organs, liver and pancreas (gastritis) |
| 117 | Past disease of digestive organs, liver and pancreas (gastric ulcer) |
| 118 | Past disease of digestive organs, liver and pancreas (duodenal ulcer) |
| 119 | Past disease of digestive organs, liver and pancreas (irritable bowel syndrome) |
| 120 | Past disease of digestive organs, liver and pancreas (Crohn's disease) |
| 121 | Past disease of digestive organs, liver and pancreas (ulcerative colitis) |
| 122 | Past disease of digestive organs, liver and pancreas (hepatic steatosis) |
| 123 | Past disease of digestive organs, liver and pancreas (hepatitis B) |
| 124 | Past disease of digestive organs, liver and pancreas (hepatitis C) |
| 125 | Past disease of digestive organs, liver and pancreas (other hepatitis) |
| 126 | Past disease of digestive organs, liver and pancreas (gallstones) |
| 127 | Past disease of digestive organs, liver and pancreas (pancreatitis) |
| 128 | Past disease of digestive organs, liver and pancreas (other) |
| 129 | Past disease of kidney (chronic nephritis (IgA nephropathy and glomerular nephritis)) |
| 130 | Past disease of kidney (nephrotic syndrome) |
| 131 | Past disease of kidney (other) |
| 132 | Past obstetric/gynecologic disease (menstrual irregularity and menoxenia (dysmenorrhea)) |
| 133 | Past obstetric/gynecologic disease (endometriosis) |
| 134 | Past obstetric/gynecologic disease (uterine myoma) |
| 135 | Past obstetric/gynecologic disease (adenomyosis uteri) |
| 136 | Past obstetric/gynecologic disease (uterine malformation) |
| 137 | Past obstetric/gynecologic disease (ovarian tumor or ovarian cyst) |
| 138 | Past obstetric/gynecologic disease (polycystic ovary syndrome) |
| 139 | Past obstetric/gynecologic disease (malformation of urinary tract or genital organs) |
| 140 | Past obstetric/gynecologic disease (other) |
| 141 | Past ophthalmologic diseases (myopia) |
| 142 | Past ophthalmologic diseases (congenital glaucoma) |
| 143 | Past ophthalmologic diseases (corneal opacity) |
| 144 | Past ophthalmologic diseases (optic atrophy) |
| 145 | Past ophthalmologic diseases (other) |
| 146 | Past disease of bone and muscle (congenital hip dislocation) |
| 147 | Past disease of bone and muscle (scoliosis) |
| 148 | Past disease of bone and muscle (other) |
| 149 | Past cancer (breast cancer) |
| 150 | Past cancer (cervical cancer) |
| 151 | Past cancer (endometrial cancer) |
| 152 | Past cancer (stomach cancer) |
| 153 | Past cancer (colorectal cancer) |
| 154 | Past cancer (blood cancer (leukemia, malignant lymphoma and multiple myeloma)) |
| 155 | Past cancer (other cancers) |
| 156 | Past developmental disorder (attention deficit and hyperactivity disorder (ADHD)) |
| 157 | Past developmental disorder (learning disability (LD)) |
| 158 | Past developmental disorder (autism, Asperger’s syndrome or pervasive developmental disorder (PDD)) |
| 159 | Past developmental disorder (other) |
| 160 | Past abnormality of pregnancy and childbirth (pregnancy-induced hypertension (pregnancy toxemia and eclampsia)) |
| 161 | Past abnormality of pregnancy and childbirth (abruption placentae) |
| 162 | Past abnormality of pregnancy and childbirth (ectopic pregnancy) |
| 163 | Past abnormality of pregnancy and childbirth (placenta previa) |
| 164 | Past abnormality of pregnancy and childbirth (hydatidiform mole) |
| 165 | Past abnormality of pregnancy and childbirth (other abnormalities of pregnancy and childbirth) |
| 166 | History of the past year (measles ) |
| 167 | History of the past year (rubella) |
| 168 | History of the past year (tuberculosis) |
| 169 | History of the past year (influenza) |
| 170 | History of the past year (mumps) |
| 171 | I have suffered from a side effect (e.g., high fever, skin rash, anaphylactic reaction etc.) after being vaccinated |
| 172 | I have received a blood transfusion |
| 173 | I took a medicine (including nonprescription drugs) or got a shot or intravenous drips (IV) during the past year |
| 174 | Frequency of taking folic acid supplement |
| 175 | How often did you feel nervous? |
| 176 | How often did you feel hopelessness? |
| 177 | How often did you feel restless or fidgety? |
| 178 | How often did you feel so depressed that nothing could cheer you up? |
| 179 | How often did you feel that everything was an effort? |
| 180 | How often did you feel worthless? |
| 181 | Overall, how would you rate your health during the past 4 weeks?, |
| 182 | How much did physical health problems limit your usual physical activities (such as walking or climbing stairs), during the past 4 weeks? |
| 183 | During the past 4 weeks, how much difficulty did you have doing your daily work, both at home and away from home, because of your physical health? |
| 184 | During the past 4 weeks, how much bodily pain have you had? |
| 185 | During the past 4 weeks, how much energy did you have? |
| 186 | During the past 4 weeks, how much did your physical health or emotional problems limit your usual social activities with family or friends? |
| 187 | During the past 4 weeks, how much have you been bothered by emotional problems (such as feeling anxious, depressed or irritable)? |
| 188 | During the past 4 weeks, how much did personal or emotional problems keep you from doing your usual work, school or other daily activities? |
| 189 | Frequency of being insulted or cursed at by husband/romantic partner prior to current pregnancy |
| 190 | Frequency of being hit or beaten up by husband/romantic partner in the course of quarrel, which led to injury, prior to current pregnancy |
| 191 | Smoking history |
| 192 | Age at starting smoking (Previously did, but quit before realizing current pregnancy) |
| 193 | Age at quitting smoking (Previously did, but quit before realizing current pregnancy) |
| 194 | Number of cigarettes smoked per day on average before quitting (Previously did, but quit before realizing current pregnancy) |
| 195 | Age at starting smoking (Previously did, but quit after realizing current pregnancy) |
| 196 | Last day of smoking: days ago (Previously did, but quit after realizing current pregnancy) |
| 197 | Number of cigarettes smoked per day on average before quitting (Previously did, but quit after realizing current pregnancy) |
| 198 | Age at starting smoking (Currently smoking) |
| 199 | Number of cigarettes smoked per day on average (Currently smoking) |
| 200 | Number of family members smoking |
| 201 | Number of individuals smoking in the rooms at home in the presence of the respondent |
| 202 | History of husband/partner (baby’s father) smoking |
| 203 | Age at starting smoking (husband/partner : previously did, but quit before recognizing current pregnancy) |
| 204 | Age at quitting smoking (husband/partner : previously did, but quit before recognizing current pregnancy) |
| 205 | Number of cigarettes smoked per day on average before quitting (husband/partner : previously did, but quit before recognizing current pregnancy) |
| 206 | Age at starting smoking (husband/partner : previously did, but quit after finding out current pregnancy) |
| 207 | Number of cigarettes smoked per day on average before quitting (husband/partner : previously did, but quit after finding out current pregnancy) |
| 208 | Age at starting smoking (husband/partner : yes, he still smokes) |
| 209 | Number of cigarettes smoked per day on average (husband/partner : yes, he still smokes) |
| 210 | Frequency of exposure to secondhand smoke at any indoor place before current pregnancy |
| 211 | Number of hours exposed to secondhand smoke per day |
| 212 | In an ordinary week before you became pregnant, how many days did you do Vigorous physical activities like heavy lifting, digging, aerobics, or fast bicycling? |
| 213 | In an ordinary week before you became pregnant, how many days did you do Vigorous physical activities like heavy lifting, digging, aerobics, or fast bicycling? (Days) |
| 214 | How much time did you usually spend doing vigorous physical activities on one of those days? (Hours, Minutes) |
| 215 | In an ordinary week before you got pregnant, how many days did you do moderate physical activities like carrying light loads, bicycling at a regular pace, doubles tennis? Do not include walking. |
| 216 | In an ordinary week before you got pregnant, how many days did you do moderate physical activities like carrying light loads, bicycling at a regular pace, doubles tennis? Do not include walking. (Days) |
| 217 | How much time did you usually spend doing moderate physical activities on one of those days? (Hours, Minutes) |
| 218 | In an ordinary week before you got pregnant, on how many days did you walk for at least 10 minutes at a time? Walking includes all the walking at work and at home, walking to travel from place to place, and any other walking that you have done solely for recreation, sport, exercise, or leisure. |
| 219 | In an ordinary week before you got pregnant, on how many days did you walk for at least 10 minutes at a time? Walking includes all the walking at work and at home, walking to travel from place to place, and any other walking that you have done solely for recreation, sport, exercise, or leisure. (Days) |
| 220 | How much time did you usually spend walking on one of those days? (Hours, Minutes) |
| 221 | In an ordinary day before you got pregnant, how much time did you spend sitting or lying down on a week day? This may include time sitting at a desk, talking with your friends, reading, or sitting or lying down to watch television. Do not include sleeping time. (Hours, Minutes) |
| 222 | Number of hours/minutes watching TV per day. (Hours, Minutes) |
| 223 | Number of hours/minutes playing videogames (including games on mobile phone/smart phone) per day. (Hours, Minutes) |
| 224 | Average bedtime before current pregnancy |
| 225 | Average bedtime before current pregnancy (Hours, Minutes) |
| 226 | Type of residence |
| 227 | Number of floors in the apartment building |
| 228 | Floor living on |
| 229 | Average wake up time before current pregnancy |
| 230 | Average wake up time before current pregnancy (Hours, Minutes) |
| 231 | Occupation before pregnancy |
| 232 | Occupation (not in the list) |
| 233 | Current employment status |
| 234 | Number of working days per week |
| 235 | Number of working hours per day (including overtime) |
| 236 | Number of night shifts per month |
| 237 | I have been engaged in at least one of the following activities after becoming pregnant |
| 238 | Lifting something weighing more than 20kg |
| 239 | Using organic solvent (e.g., paint thinner, glue, etc.) |
| 240 | Being suffered from loud noise |
| 241 | Using manufacturing tools with vibration |
| 242 | Going in and out of commercial refrigerator or freezer |
| 243 | Working at a hot place making one sweaty |
| 244 | Working in a dusty environment |
| 245 | Frequency of using or handling following materials during work for more than half day. a. Kerosene, Petroleum, Benzene, Gasoline |
| 246 | Frequency of using or handling following materials during work for more than half day. b. Chlorine bleach, Germicide (with the warning, “Do not mix. Hazardous.”) |
| 247 | Frequency of using or handling following materials during work for more than half day. c. Medical disinfectant |
| 248 | Frequency of using or handling following materials during work for more than half day. d. Permanent marker |
| 249 | Frequency of using or handling following materials during work for more than half day. e. Water-based paint or Inkjet printer |
| 250 | Frequency of using or handling following materials during work for more than half day. f. Organic solvents (e.g., paint thinner, solvent for examination/analysis/extraction, dry-cleaning detergent, stain-removing agent, paint coating, nail polish remover, etc.) |
| 251 | Frequency of using or handling following materials during work for more than half day. g. Photocopier, Laser printer |
| 252 | Frequency of using or handling following materials during work for more than half day. h. Engine oil |
| 253 | Frequency of using or handling following materials during work for more than half day. i. Formalin, Formaldehyde |
| 254 | Frequency of using or handling following materials during work for more than half day. j. Anticancer drug (excluding those prescribed to the respondent) |
| 255 | Frequency of using or handling following materials during work for more than half day. k. General anesthetic for surgery |
| 256 | Frequency of using or handling following materials during work for more than half day. l. Insecticide |
| 257 | Frequency of using or handling following materials during work for more than half day. m. Herbicide |
| 258 | Frequency of using or handling following materials during work for more than half day. n. Unidentified or other agricultural chemical |
| 259 | Frequency of using or handling following materials during work for more than half day. o. Radiation, Radioactive substances, Isotopes |
| 260 | Frequency of using or handling following materials during work for more than half day. p. Microbes |
| 261 | Frequency of using or handling following materials during work for more than half day. q. Any products containing lead (e.g., solder) |
| 262 | Frequency of using or handling following materials during work for more than half day. r. Lead-free solder |
| 263 | Frequency of using or handling following materials during work for more than half day. s. Chromium, Arsenic, Cadmium |
| 264 | Frequency of using or handling following materials during work for more than half day. t. Mercury |
| 265 | Frequency of using or handling following materials during work for more than half day.U. Dyestuffs (for hair coloring) |
| 266 | Frequency of using or handling following materials during work for more than half day. v. Other chemical substances |
| 267 | I took medicine/drug/supplements in the past year |
| 268 | I took medicine/drug/supplement. Since one year ago before pregnancy was found out |
| 269 | I took medicine/drug/supplement. During the first trimester |
| 270 | I took medicine/drug/supplement. Between the beginning of the second trimester and now |
| 271 | Highest level of education |
| 272 | Partner's highest level of education |
| 273 | Annual household income |
| 274 | Number of individuals in family who contribute to household income |
| 275 | Mother's birth weight (approximately ____g) |
| 276 | Father's birth weight (approximately ____g) |
| 277 | Age of mother |
| 278 | Weeks and day(s) of pregnancy at the time of enrollment. (Weeks) |
| 279 | Weeks and day(s) of pregnancy at the time of enrollment. (Days) |
| 280 | Method used for calculating expected date of delivery |
| 281 | Expected place for delivery |
| 282 | Mother's current weight |
| 283 | Year of measuring height and weight |
| 284 | Month of measuring height and weight |
| 285 | How did the mother become pregnant this time? |
| 286 | Number of fetus and chorionicity |
| 287 | Have menstrual abnormality |
| 288 | Menstrual abnormality (unusually short menstruation) |
| 289 | Menstrual abnormality (unusually long menstruation) |
| 290 | Menstrual abnormality (hypomenorrhea) |
| 291 | Menstrual abnormality (hypermenorrhea) |
| 292 | Menstrual abnormality (polycystic Ovary Syndrome) |
| 293 | Menstrual abnormality (menstrual difficulty (light)) |
| 294 | Menstrual abnormality (menstrual difficulty (severe)) |
| 295 | Menstrual abnormality (other type of menstrual abnormality) |
| 296 | Regular medication in the past year |
| 297 | Hysterosalpingogram test administered within the three month before current pregnancy |
| 298 | Number of previous pregnancies |
| 299 | Number of previous deliveries |
| 300 | 1st pregnancy Result of pregnancy |
| 301 | 1st pregnancy Age of mother at the delivery |
| 302 | 1st pregnancy Pregnancy period (number of weeks) |
| 303 | 1st pregnancy Pregnancy period (full-term or not) |
| 304 | 1st pregnancy Number of fetus |
| 305 | 1st pregnancy Sex of the baby |
| 306 | 1st pregnancy Birthweight |
| 307 | 1st pregnancy Result of pregnancy (alive or dead) |
| 308 | 1st pregnancy Abnormal pregnancy complications |
| 309 | 1st pregnancy Pregnancy diabetes |
| 310 | 1st pregnancy Placental abruption |
| 311 | 1st pregnancy Ectopic pregnancy |
| 312 | 1st pregnancy Placenta previa |
| 313 | 1st pregnancy Hydatidiform mole |
| 314 | 1st pregnancy Other pregnancy complication |
| 315 | 2nd pregnancy Result of pregnancy |
| 316 | 2nd pregnancy Age of mother at the delivery |
| 317 | 2nd pregnancy Pregnancy period (number of weeks) |
| 318 | 2nd pregnancy Pregnancy period (full-term or not) |
| 319 | 2nd pregnancy Number of fetus |
| 320 | 2nd pregnancy Sex of the baby |
| 321 | 2nd pregnancy Birthweight |
| 322 | 2nd pregnancy Result of pregnancy (alive or dead) |
| 323 | 2nd pregnancy Abnormal pregnancy complications |
| 324 | 2nd pregnancy Pregnancy diabetes |
| 325 | 2nd pregnancy Placental abruption |
| 326 | 2nd pregnancy Ectopic pregnancy |
| 327 | 2nd pregnancy Placenta previa |
| 328 | 2nd pregnancy Hydatidiform mole |
| 329 | 2nd pregnancy Other pregnancy complication |
| 330 | 3rd pregnancy Result of pregnancy |
| 331 | 3rd pregnancy Age of mother at the delivery |
| 332 | 3rd pregnancy Pregnancy period (number of weeks) |
| 333 | 3rd pregnancy Pregnancy period (full-term or not) |
| 334 | 3rd pregnancy Number of fetus |
| 335 | 3rd pregnancy Sex of the baby |
| 336 | 3rd pregnancy Birthweight |
| 337 | 3rd pregnancy Result of pregnancy (alive or dead) |
| 338 | 3rd pregnancy Abnormal pregnancy complications |
| 339 | 3rd pregnancy Pregnancy diabetes |
| 340 | 3rd pregnancy Placental abruption |
| 341 | 3rd pregnancy Ectopic pregnancy |
| 342 | 3rd pregnancy Placenta previa |
| 343 | 3rd pregnancy Hydatidiform mole |
| 344 | 3rd pregnancy Other pregnancy complication |
| 345 | 4th pregnancy Result of pregnancy |
| 346 | 4th pregnancy Age of mother at the delivery |
| 347 | 4th pregnancy Pregnancy period (number of weeks) |
| 348 | 4th pregnancy Pregnancy period (full-term or not) |
| 349 | 4th pregnancy Number of fetus |
| 350 | 4th pregnancy Sex of the baby |
| 351 | 4th pregnancy Birthweight |
| 352 | 4th pregnancy Result of pregnancy (alive or dead) |
| 353 | 4th pregnancy Abnormal pregnancy complications |
| 354 | 4th pregnancy Pregnancy diabetes |
| 355 | 4th pregnancy Placental abruption |
| 356 | 4th pregnancy Ectopic pregnancy |
| 357 | 4th pregnancy Placenta previa |
| 358 | 4th pregnancy Hydatidiform mole |
| 359 | 4th pregnancy Other pregnancy complication |
| 360 | 5th pregnancy Result of pregnancy |
| 361 | 5th pregnancy Age of mother at the delivery |
| 362 | 5th pregnancy Pregnancy period (number of weeks) |
| 363 | 5th pregnancy Pregnancy period (full-term or not) |
| 364 | 5th pregnancy Number of fetus |
| 365 | 5th pregnancy Sex of the baby |
| 366 | 5th pregnancy Birthweight |
| 367 | 5th pregnancy Result of pregnancy (alive or dead) |
| 368 | 5th pregnancy Abnormal pregnancy complications |
| 369 | 5th pregnancy Pregnancy diabetes |
| 370 | 5th pregnancy Placental abruption |
| 371 | 5th pregnancy Ectopic pregnancy |
| 372 | 5th pregnancy Placenta previa |
| 373 | 5th pregnancy Hydatidiform mole |
| 374 | 5th pregnancy Other pregnancy complication |
| 375 | 6th pregnancy Result of pregnancy |
| 376 | 6th pregnancy Age of mother at the delivery |
| 377 | 6th pregnancy Pregnancy period (number of weeks) |
| 378 | 6th pregnancy Pregnancy period (full-term or not) |
| 379 | 6th pregnancy Number of fetus |
| 380 | 6th pregnancy Sex of the baby |
| 381 | 6th pregnancy Birthweight |
| 382 | 6th pregnancy Result of pregnancy (alive or dead) |
| 383 | 6th pregnancy Abnormal pregnancy complications |
| 384 | 6th pregnancy Pregnancy diabetes |
| 385 | 6th pregnancy Placental abruption |
| 386 | 6th pregnancy Ectopic pregnancy |
| 387 | 6th pregnancy Placenta previa |
| 388 | 6th pregnancy Hydatidiform mole |
| 389 | 6th pregnancy Other pregnancy complication |
| 390 | 7th pregnancy Result of pregnancy |
| 391 | 7th pregnancy Age of mother at the delivery |
| 392 | 7th pregnancy Pregnancy period (number of weeks) |
| 393 | 7th pregnancy Pregnancy period (full-term or not) |
| 394 | 7th pregnancy Number of fetus |
| 395 | 7th pregnancy Sex of the baby |
| 396 | 7th pregnancy Birthweight |
| 397 | 7th pregnancy Result of pregnancy (alive or dead) |
| 398 | 7th pregnancy Abnormal pregnancy complications |
| 399 | 7th pregnancy Pregnancy diabetes |
| 400 | 7th pregnancy Placental abruption |
| 401 | 7th pregnancy Ectopic pregnancy |
| 402 | 7th pregnancy Placenta previa |
| 403 | 7th pregnancy Hydatidiform mole |
| 404 | 7th pregnancy Other pregnancy complication |
| 405 | 8th pregnancy Result of pregnancy |
| 406 | 8th pregnancy Age of mother at the delivery |
| 407 | 8th pregnancy Pregnancy period (number of weeks) |
| 408 | 8th pregnancy Pregnancy period (full-term or not) |
| 409 | 8th pregnancy Number of fetus |
| 410 | 8th pregnancy Sex of the baby |
| 411 | 8th pregnancy Birthweight |
| 412 | 8th pregnancy Result of pregnancy (alive or dead) |
| 413 | 8th pregnancy Abnormal pregnancy complications |
| 414 | 8th pregnancy Pregnancy diabetes |
| 415 | 8th pregnancy Placental abruption |
| 416 | 8th pregnancy Ectopic pregnancy |
| 417 | 8th pregnancy Placenta previa |
| 418 | 8th pregnancy Hydatidiform mole |
| 419 | 8th pregnancy Other pregnancy complication |
| 420 | Recurrent miscarriage |
| 421 | Cause of recurrent miscarriage: Unknown |
| 422 | Cause of recurrent miscarriage:antiphospholipid antibody syndrome |
| 423 | Cause of recurrent miscarriage:uterine deformity |
| 424 | Cause of recurrent miscarriage:chromosomal abnormality of parent |
| 425 | Cause of recurrent miscarriage: others |
| 426 | Age at which chromosomal abnormality of parents was found |
| 427 | Age at which other cause of recurrent miscarriage was found |
| 428 | Stable HbA1c (HPLC) |
| 429 | IgE measurement (Dust mite) |
| 430 | IgE measurement (Dust mite : Specific IgE antibody titer) |
| 431 | IgE measurement (Cedar) |
| 432 | IgE measurement (Cedar : Specific IgE antibody titer) |
| 433 | IgE measurement (Egg white) |
| 434 | IgE measurement (Egg white : Specific IgE antibody titer) |
| 435 | IgE measurement (Specific IgE (multi-allergen) epithelium) |
| 436 | IgE measurement (Specific IgE (multi-allergen) epithelium : Specific IgE antibody titer) |
| 437 | IgE measurement (Moth) |
| 438 | IgE measurement (Moth ; Specific IgE antibody titer) |
| 439 | IgE measurement (Birch) |
| 440 | IgE measurement (Birch : Specific IgE antibody titer) |
| 441 | Total IgE |
| 442 | White blood cell count |
| 443 | Red blood cell count |
| 444 | Hematocrit |
| 445 | Hematocrit value |
| 446 | Mean corpuscular volume |
| 447 | Mean corpuscular hemorrhage |
| 448 | Mean corpuscular hemoglobin concentration |
| 449 | Platelet count |
| 450 | Neutrophils |
| 451 | Lymphocytes |
| 452 | Monocyte |
| 453 | Eosinophil |
| 454 | Basophil |
| 455 | Metamyelocytes |
| 456 | Atypical-lymphocyte |
| 457 | Myelocyte |
| 458 | Myeloblast |
| 459 | Promyelocyte |
| 460 | Erythroblast |
| 461 | Plasma cell |
| 462 | LDL cholesterol quantification |
| 463 | Total cholesterol |
| 464 | Free cholesterol |
| 465 | Triglyceride |
| 466 | HDL-Cholesterol |
| 467 | Total protein |
| 468 | Albumin quantification |
| 469 | IGF-I |
| 470 | Phospholipid |
| 471 | Urinary Creatinine |
| 472 | Urine specific gravity |
| 473 | FFQ Has your eating habits changed significantly within the last five years? |
| 474 | FFQ Why has it changed? |
| 475 | FFQ Are you the one who just eats too much? |
| 476 | FFQ How fast do you eat? |
| 477 | FFQ How often do you eat "breakfast"? |
| 478 | FFQ How often do you "eat out"? (Bento and rice balls bought at the store are counted as eating out) |
| 479 | FFQ How often do you eat "convenience foods"? (Ramen, cup noodles, retort foods, etc.) |
| 480 | FFQ How often do you eat "stir-fried foods (stir-fried vegetables, etc.)" with oil? |
| 481 | FFQ How often do you eat oiled "fried foods (fried foods, tempura, etc.)"? |
| 482 | FFQ How much do you eat meat oil? |
| 483 | FFQ How much do you drink ramen, udon, and soba soup? |
| 484 | FFQ Do you have a habit of sprinkling salt on your dishes at the table? |
| 485 | FFQ Do you have a habit of sprinkling soy sauce on your dishes at the table? |
| 486 | FFQ Please select the oil you use most often and check only one. |
| 487 | FFQ Please check only one of the most common cooking methods. What about meat? |
| 488 | FFQ What about fish and shellfish? |
| 489 | FFQ What about vegetables? |
| 490 | FFQ How do you eat steak and yakiniku most often? |
| 491 | FFQ How often do you eat "grilled fish"? |
| 492 | FFQ When you eat "grilled fish", do you eat the burnt part? |
| 493 | FFQ How do you like your food? Choose one of the following: What is the rich" dish? " |
| 494 | FFQ What is the "Karai taste" dish? |
| 495 | FFQ What is the "deep taste of salt" dish? |
| 496 | FFQ What is the "sour taste" dish? |
| 497 | FFQ What about sweets such as sweets? |
| 498 | FFQ What about hot food and drinks? |
| 499 | FFQ We will ask you about "rice (rice)". How big do you eat in a bowl? |
| 500 | FFQ How many cups do you eat a day, including morning, lunch and dinner? |
| 501 | FFQ What is the average percentage of brown rice in a day's rice? |
| 502 | FFQ Do you mix "wheat"? |
| 503 | FFQ Do you mix "Awa / Hie"? |
| 504 | FFQ We will ask you about "miso soup". How often do you drink? |
| 505 | FFQ How many cups do you drink a day for morning, lunch and dinner? |
| 506 | FFQ What kind of seasoning is it? |
| 507 | FFQ Energy (kcal) |
| 508 | FFQ Water (g) |
| 509 | FFQ Protein (g) |
| 510 | FFQ Lipid (g) |
| 511 | FFQ Carbohydrate (g) |
| 512 | FFQ Ash content (g) |
| 513 | FFQ Sodium (mg) |
| 514 | FFQ Potassium (mg) |
| 515 | FFQ Calcium (mg) |
| 516 | FFQ Magnesium (mg) |
| 517 | FFQ Phosphorus (mg) |
| 518 | FFQ Iron (mg) |
| 519 | FFQ Zinc (mg) |
| 520 | FFQ Copper (mg) |
| 521 | FFQ Manganese (mg) |
| 522 | FFQ Retinol (μg) |
| 523 | FFQ Retinol equivalent (μg) |
| 524 | FFQ α-carotene (μg) |
| 525 | FFQ β-carotene (μg) |
| 526 | FFQ Cryptoxanthin (μg) |
| 527 | FFQ β-carotene equivalent (μg) |
| 528 | FFQ Vitamin D (μg) |
| 529 | FFQ α-tocopherol (mg) |
| 530 | FFQ β-tocopherol (mg) |
| 531 | FFQ γ-tocopherol (mg) |
| 532 | FFQ δ-Tocopherol (mg) |
| 533 | FFQ Vitamin K (μg) |
| 534 | FFQ Vitamin B1 (mg) |
| 535 | FFQ Vitamin B2 (mg) |
| 536 | FFQ Niacin (mg) |
| 537 | FFQ Vitamin B6 (mg) |
| 538 | FFQ Vitamin B12 (μg) |
| 539 | FFQ Folic acid (μg) |
| 540 | FFQ Pantothenic acid (mg) |
| 541 | FFQ Vitamin C (mg) |
| 542 | FFQ Saturated fatty acids (g) |
| 543 | FFQ Monounsaturated fatty acids (g) |
| 544 | FFQ Polyunsaturated fatty acids (g) |
| 545 | FFQ Cholesterol (mg) |
| 546 | FFQ Water-soluble dietary fiber (g) |
| 547 | FFQ Insoluble dietary fiber (g) |
| 548 | FFQ Total amount of dietary fiber (g) |
| 549 | FFQ Salt equivalent (g) |
| 550 | FFQ Dijain (mg) |
| 551 | FFQ Genistane (mg) |
| 552 | FFQ Lycopene (μg) |
| 553 | FFQ n-3 unsaturated fatty acids (g) |
| 554 | FFQ n-6 unsaturated fatty acids (g) |
| 555 | FFQ Selenium (μg) |
| 556 | FFQ Protein by amino acid composition (g) |
| 557 | FFQ Triacylglycerol equivalent (g) |
| 558 | FFQ Iodine (μg) |
| 559 | FFQ Chromium (μg) |
| 560 | FFQ Molybdenum (μg) |
| 561 | FFQ Biotin (μg) |
| 562 | FFQ Ethanol (g / d) |
| 563 | FFQ Energy (kj) |
| 564 | FFQ Sake |
| 565 | FFQ Shochu / Awamori |
| 566 | FFQ Large beer bottle |
| 567 | FFQ Whiskey |
| 568 | FFQ Wine |
| 569 | FFQ Rice |
| 570 | FFQ Brown rice |
| 571 | FFQ Wheat |
| 572 | FFQ Awa / Hie |
| 573 | FFQ Miso soup |
| 574 | FFQ Beef steak |
| 575 | FFQ Beef fried food (grilled meat, etc.) |
| 576 | FFQ Stir-fried beef (stir-fried vegetables, etc.) |
| 577 | FFQ Stewed beef (curry, stew, etc.) |
| 578 | FFQ Stir-fried pork (stir-fried vegetables, etc.) |
| 579 | FFQ Fried pork (pork cutlet, etc.) |
| 580 | FFQ Stewed pork (curry, stew, etc.) |
| 581 | FFQ Boiled pork (Kakuni, Okinawa name Rafute, etc.) |
| 582 | FFQ Pork soup (pork soup, Okinawan nakami soup, etc.) |
| 583 | FFQ Pork Pork liver (stir-fried Nirareba, etc.) |
| 584 | FFQ Grilled chicken (yakitori, etc.) |
| 585 | FFQ Stir-fried chicken (stir-fried vegetables, etc.) |
| 586 | FFQ Boiled chicken |
| 587 | FFQ Fried chicken (fried chicken, etc.) |
| 588 | FFQ Chicken chicken liver (yakitori, etc.) |
| 589 | FFQ Loin ham |
| 590 | FFQ Wiener sausage |
| 591 | FFQ Bacon |
| 592 | FFQ Canned luncheon meat |
| 593 | FFQ Milk low fat milk |
| 594 | FFQ Milk normal milk |
| 595 | FFQ Egg |
| 596 | FFQ Cheese |
| 597 | FFQ Yogurt |
| 598 | FFQ Salted atka mackerel, salted atka mackerel, salted salmon |
| 599 | FFQ Strings (such as horse mackerel opening) |
| 600 | FFQ Canned tuna (sea chicken flakes) |
| 601 | FFQ Salmon |
| 602 | FFQ Skipjack and tuna |
| 603 | FFQ Yellowtail / Hamachi |
| 604 | FFQ Flatfish |
| 605 | FFQ Taikin (red sea bream, Okinawa name Gurukun, Okinawa name town, etc.) |
| 606 | FFQ Horse mackerel and horse mackerel |
| 607 | FFQ pacific saury mackerel |
| 608 | FFQ Shirasuboshi |
| 609 | FFQ Cod roe, sujiko, salmon roe |
| 610 | FFQ Eel |
| 611 | FFQ Squid |
| 612 | FFQ Octopus |
| 613 | FFQ Shrimp |
| 614 | FFQ Asari / Shijimi |
| 615 | FFQ Snail |
| 616 | FFQ Chikuwa |
| 617 | FFQ Kamaboko |
| 618 | FFQ Satsuma-age |
| 619 | FFQ Carrots |
| 620 | FFQ Spinach |
| 621 | FFQ Pumpkin |
| 622 | FFQ Cabbage |
| 623 | FFQ Radish |
| 624 | FFQ Pickles Takuwan |
| 625 | FFQ Pickles Green leaf pickles (Nozawana, Takana) |
| 626 | FFQ Pickled Umeboshi |
| 627 | FFQ Pickles are Chinese cabbage |
| 628 | FFQ Pickled cucumber |
| 629 | FFQ Pickled eggplant |
| 630 | FFQ Pickled turnip |
| 631 | FFQ Green pepper |
| 632 | FFQ Tomato |
| 633 | FFQ Leek |
| 634 | FFQ Green onions and scallions |
| 635 | FFQ Garlic chives |
| 636 | FFQ Crowndaisy |
| 637 | FFQ Komatsuna |
| 638 | FFQ Broccoli |
| 639 | FFQ Onion |
| 640 | FFQ Cucumber |
| 641 | FFQ Eggplant |
| 642 | FFQ Chinese cabbage |
| 643 | FFQ Burdock |
| 644 | FFQ Bean sprouts |
| 645 | FFQ Green beans |
| 646 | FFQ Lettuce Chisha |
| 647 | FFQ Green asparagus |
| 648 | FFQ Garlic |
| 649 | FFQ Bok choy (Okinawa name Pakuchoi) |
| 650 | FFQ Mustard greens (Okinawa name Shimaner) |
| 651 | FFQ Bitter melon (Okinawa name bitter gourd) |
| 652 | FFQ Usually so (Okinawa name sbanner) |
| 653 | FFQ Loofah (Okinawa name Nabera) |
| 654 | FFQ Mugwort (Okinawa name Fuchiba) |
| 655 | FFQ Papaya |
| 656 | FFQ Mandarin orange |
| 657 | FFQ Other citrus fruits (Hassaku, Iyokan, Orange) |
| 658 | FFQ Apple |
| 659 | FFQ Persimmon |
| 660 | FFQ Strawberry |
| 661 | FFQ Grapes |
| 662 | FFQ Melon |
| 663 | FFQ Watermelon |
| 664 | FFQ Peaches |
| 665 | FFQ None |
| 666 | FFQ Kiwi fruit |
| 667 | FFQ Pineapple |
| 668 | FFQ Banana |
| 669 | FFQ Mango |
| 670 | FFQ Breads (including sweet buns) |
| 671 | FFQ Udon |
| 672 | FFQ Soba |
| 673 | FFQ Okinawa soba |
| 674 | FFQ Ramen |
| 675 | FFQ Pasta (spaghetti, macaroni, etc.) |
| 676 | FFQ Somen / Hiyamugi |
| 677 | FFQ Rice cake |
| 678 | FFQ Japanese sweets (Daifuku Manju) |
| 679 | FFQ Cake |
| 680 | FFQ Biscuits and cookies |
| 681 | FFQ Chocolate |
| 682 | FFQ Ice cream |
| 683 | FFQ Snacks (potato chips, etc.) |
| 684 | FFQ Senbei |
| 685 | FFQ Sesame |
| 686 | FFQ Peanuts and peanuts |
| 687 | FFQ Tofu (miso soup ingredient) |
| 688 | FFQ Tofu (Yudofu, Hiyayakko, etc.) |
| 689 | FFQ Yushi Dofu (Oboro Dofu) |
| 690 | FFQ Takano Dofu / Shimitoufu |
| 691 | FFQ Atsuage / Atsuage |
| 692 | FFQ Aburaage |
| 693 | FFQ Natto |
| 694 | FFQ Sweet potato |
| 695 | FFQ Potatoes |
| 696 | FFQ Taro |
| 697 | FFQ Yam and long |
| 698 | FFQ Konjac / Shirataki |
| 699 | FFQ Shiitake |
| 700 | FFQ Enoki mushroom |
| 701 | FFQ Shimeji mushroom |
| 702 | FFQ Wakame seaweed, kelp |
| 703 | FFQ Hijiki |
| 704 | FFQ Nori (grilled seaweed / seasoned seaweed) |
| 705 | FFQ Butter on bread |
| 706 | FFQ Margarine on bread |
| 707 | FFQ Jam marmalade on bread |
| 708 | FFQ Honey |
| 709 | FFQ Dressing |
| 710 | FFQ Mayonnaise |
| 711 | FFQ Source |
| 712 | FFQ Ketchup |
| 713 | FFQ Mustard |
| 714 | FFQ Wasabi |
| 715 | FFQ Chili pepper |
| 716 | FFQ Ginger |
| 717 | FFQ Kinako |
| 718 | FFQ Japanese tea (made from leaves) |
| 719 | FFQ Japanese tea (cans and PET bottles) |
| 720 | FFQ Oolong tea (from leaves) |
| 721 | FFQ Oolong tea (cans / PET bottles) |
| 722 | FFQ Black tea (from leaves) |
| 723 | FFQ Tea (cans / PET bottles) |
| 724 | FFQ Coffee (from beans) |
| 725 | FFQ Coffee (instant) |
| 726 | FFQ Coffee (cans / PET bottles) |
| 727 | FFQ Tomato juice |
| 728 | FFQ Vegetable juice |
| 729 | FFQ 100% fruit juice orange juice |
| 730 | FFQ 100% fruit juice apple juice |
| 731 | FFQ 100% fruit juice grapefruit juice |
| 732 | FFQ Fruit juice drink (juice that is not 100%) |
| 733 | FFQ Carbonated drinks |
| 734 | FFQ Soy milk |
| 735 | FFQ Lactic acid bacteria beverage (Yakult, etc.) |
| 736 | FFQ Drinking water (tap water / well water) |
| 737 | FFQ Drinking water (commercially available / water purifier) |
| 738 | FFQ Black tea sugar |
| 739 | FFQ Tea milk |
| 740 | FFQ Coffee sugar |
| 741 | FFQ Coffee milk |
| 742 | FFQ Cooking salt |
| 743 | FFQ Men soup |
| 744 | FFQ Salad oil (blended oil) |
| 745 | FFQ Safflower oil (safflower oil) |
| 746 | FFQ Corn oil |
| 747 | FFQ Soybean oil |
| 748 | FFQ Canola oil / canola oil |
| 749 | FFQ Olive oil |
| 750 | FFQ others |
| 751 | FFQ Cereals |
| 752 | FFQ Potatoes and starches |
| 753 | FFQ Sugar and sweets |
| 754 | FFQ Beans |
| 755 | FFQ Nuts and seeds |
| 756 | FFQ Vegetables |
| 757 | FFQ Pickles |
| 758 | FFQ Green and yellow vegetables |
| 759 | FFQ Other vegetables |
| 760 | FFQ Fruits |
| 761 | FFQ Mushrooms |
| 762 | FFQ Algae |
| 763 | FFQ seafood |
| 764 | FFQ meat |
| 765 | FFQ Eggs |
| 766 | FFQ Milk |
| 767 | FFQ Oils and fats |
| 768 | FFQ Confectionery |
| 769 | FFQ Good drinks (alcohol) |
| 770 | FFQ Good drinks (other than alcohol) |
| 771 | FFQ Seasonings and spices |
| 772 | FFQ Water |
| 773 | FFQ Juice |
| 774 | FFQ Ethanol (per week) |
| 775 | FFQ How often do you drink alcohol |

SF-8 PCS, SF-8 physical component summary; SF-8 MCS, SF-8 mental component summary;
IPAQ, International Physical Activity Questionnaire;
K6, Kessler Psychological Distress Scale; METs, metabolic equivalents;
IgE, immunogloblin E; HDL-Cholesterol, High Density Lipoprotein-Cholesterol;
FFQ, food frequency questionnaires
